# Barriers and facilitators to adherence to walking group exercise in older people living with dementia in the community: a systematic review

**DOI:** 10.1186/s11556-020-00246-6

**Published:** 2020-09-21

**Authors:** J. Vseteckova, K. Dadova, R. Gracia, G. Ryan, E. Borgstrom, J. Abington, M. Gopinath, Y. Pappas

**Affiliations:** 1 Faculty of Wellbeing, Education and Language Studies, The Open University, Milton Keynes, MK7 6AA UK; 2 Faculty of Physical Education and Sport, Charles University, Prague, Czech Republic; 3 Institute for Health Research, University of Bedfordshire, Bedford, UK

**Keywords:** Adherence, Barriers, Facilitators, Walking group exercise, Ageing, Dementia

## Abstract

**Background & Aims:**

Evidence suggests that targeted exercise is important for people living with dementia. The aim of this review was to collect and synthesize evidence on the known barriers and facilitators to adherence to walking group exercise of older people living with dementia in the community.

**Methods:**

We have searched appropriate electronic databases between January 1990 until September 2019, in any language. Additionally, we searched trial registries (clinicaltrial.gov and WHO ICTRP) for ongoing studies. We included all study designs. Studies were excluded when participants were either healthy older people or people suffering from dementia but living in residential care. Narrative synthesis was used.

**Findings:**

10 papers met the inclusion criteria. The narrative analysis focused on barriers, facilitators, and adherence. All studies reported on barriers and facilitators. Barriers included: bio-medical reasons (including mental wellbeing and physical ability); relationship dynamics; and socio-economic reasons and environmental issues. Facilitators included: bio-medical benefits & benefits related to physical ability; staff, group relationship dynamics and social aspect of walking group; environmental issues and individual tailoring; and participants perceptions about the walks & the program. Most studies did not provide data about adherence or attendance; where reported, adherence ranged from 47 to 89%.

**Conclusions:**

This systematic review of literature has highlighted known barriers and facilitators to adherence to walking groups type of exercise for people living with dementia in community. Carers’ willingness to engage, their circumstances, perspectives and previous experiences of exercise seem to play a key role in facilitating adherence but there is little research that explores these. Also, the design, location and organisation of walking groups facilitate adherence. This reflects the need for such activities to be part of a wider ‘program of care’, tailored to the needs of the individual, flexible and convenient. Knowledgeable and well-trained instructors or healthcare professionals are recommended as group exercise leaders.

## Introduction

Walking is a particularly accessible form of exercise, especially for older people [1, 2]. Some research suggests that compared to high intensity exercise interventions, people are more likely to adhere to moderate forms of exercise interventions, such as, walking [3]. Growing evidence suggests a range of benefits from both targeted group walking and outdoor walking group activity for community dwelling people with mild to moderate dementia. Participation in targeted group walking programs is shown to improve memory and attention (4); and improved functional and cognitive ability (5). Other benefits from outdoor walking group activities include: continuity of self and identity [4–6]; raised self-esteem, pleasure derived from observing nature, spatial awareness, mood upliftment, reduced agitation, relaxation, improved communication and, enjoyment from sensory stimulation [7, 8]. Physical and psychological benefits apart, walking as part of a group can enable people with dementia to become (or remain) part of social networks [9].

Despite cognitive declines, participation in meaningful activities remains important for people with dementia [10]. Notably, research suggests that with progressive impairment, people with dementia are known to prefer walking to other forms of physical activity [5], highlighting the accessibility and acceptability of walking. Not unlike other people, being outdoors in nature also remains valued [11–13]. Walking may be done independently but over time family and friends, often known as informal carers [14], are important sources of social support for people with dementia to promote, facilitate and sustain engagement and participation in physical activity [15]. Some studies also indicate that over and above to the opportunities for social engagement and companionship, group walking interventions are a likely motivator for participation and adherence [16] and especially amongst older adults [17].

Considering the emerging evidence about the broad range of wellbeing benefits of group walking, including benefits to cognitive function and the significance of walking for people with dementia, research and practice is beginning to suggest the usefulness of community-based group walking interventions for people with dementia [5, 12]. However, the evidence about facilitators and barriers to group walking interventions amongst community dwelling people with dementia is limited [18].

Developing knowledge about facilitators and barriers is important as it is well established that adherence to physical activity remains variable amongst older people [19], despite widespread knowledge of health and wellbeing benefits and significance of sustained participation for realizing benefits of physical activity [20]. Adherence is usually described to denote sustained participation and as the percentage of people who finish the entire exercise program [21]. Evidence indicates a range of social, cognitive, physiological and environmental barriers and facilitators that influence adherence to physical activity amongst older adults in both community and residential care settings [22–24]. For people with dementia, who are more likely to be physically and socially less active, progressive cognitive impairment is likely to introduce additional and unique challenges.

Therefore, the purpose of this systematic review is to investigate existing literature to identify known facilitators and barriers shaping adherence to group walking programs amongst community dwelling older adults with dementia. This is articulated through the following research question - *What are the known barriers and facilitators shaping adherence to group walking programmes amongst community dwelling older adults with dementia?*’ In doing so, we aim to extend and contribute new knowledge to inform and support development of effective walking group interventions for community living people with dementia. Given that health and wellbeing of people with dementia is increasingly being recognized and prioritized in the UK and International policy contexts, our review is timely and relevant to enable people with dementia to stay active.

## Material and methods

A systematic literature search was applied and PRISMA criteria were followed in assessing and reporting. Literature was scoped to identify the most relevant terms in what seems to be a broad spectrum of participants and interventions related to barriers and facilitators to adherence to finally focus on walking group type of exercise in older people living with dementia in the community. The sub-sections below outline in more detail our search strategy, inclusion/exclusion criteria, and analysis.

Heterogeneity of outcomes and other PICO (population, intervention, control, and outcomes) criteria were assessed. Heterogeneity was found to be high, therefore a narrative synthesis approach was used, using thematic analysis for categorising data. Narrative synthesis is a commonly used method to synthesise data in the context of a systematic review [25–27]. This approach has been used by the authors successfully in the past [24, 28, 29], especially as we included and appraised mixed methods papers: systematic review, qualitative, quantitative and mixed studies and grey literature. Thematic analysis provided the means of identifying relevant themes (based on the review question) across large and diverse bodies of research [30–32]. The PICO framework was used for framing the inclusion and exclusion criteria (see below).

- **Participants:** older people living with dementia in the community, worldwide
- **Intervention:** walking group exercise, both indoor and outdoor
- **Control:** not applicable
- **Outcomes:** Barriers and facilitators to adherence to specific interventions (walking group exercise activity in improving physical, social and mental wellbeing of people living with dementia), attendance rates & dropout rates (where available); main focus on barriers and facilitators to adherence.

### Search for literature

We conducted electronic searches using the following databases: MEDLINE (Ovid), The Cochrane Central Register of Controlled Trials (CENTRAL) (Wiley), PsychINFO (Ovid), Educational Resource Information Centre (ERIC) (Ovid), Cumulative Index to Nursing and Allied Health Literature (CINAHL) (Ebsco), Web of Science Core Collection (Thomson Reuters), Trial registries (clinicaltrial.gov and WHO ICTRP) search for ongoing studies, SCOPUS, Google Scholar, and Web of science.

We devised a search strategy with keywords: Dementia, Alzheimer*, cognitive* impairment, memory loss, mild cognitive impairment AND home and /or community dwelling AND walk*, walking group, rambl* AND adher*, complian*, engag*, continuous engag* AND barrier*, obstacles, challeng*, facilitat*.

Databases were searched from January 1990 to the present (the last searches were conducted in November 2019 to make sure no new papers were published as the works on this review progressed). For all included studies, we searched reference lists. We also searched the list of references of other relevant systematic reviews identified whilst running the electronic searches.

### Types of studies

The searches were not limited to a specific study design. Hence, all types of study designs - any type of qualitative, quantitative, and mixed-methods studies - were included as long as they focused on evaluating the barriers and facilitators to adherence of walking group exercise activity in improving physical, social and mental wellbeing of people living with dementia. We were also looking for studies to mention/discuss adherence wherever possible. From our experience [32] not all authors have or present data on adherences. Apart from qualitative studies, a whole range of quantitative studies were included in our searches such as randomised, cluster-randomised or quasi-randomised controlled trials, cohort studies, before-and-after studies and interrupted time series studies. Journal articles as well as conference proceedings were included in the searches. We have also searched for and included grey literature.

### Other criteria

Studies from around the world were included as long as an abstract and the paper were written/available in English.

Studies not reporting on participation in walking exercise group activities in older people living with dementia in the community were excluded. Studies not reporting on barriers and facilitators to adherence to such exercise were excluded. Due to limited evidence available, studies were included if participants suffered from any forms of dementia.

### Selection of studies

Titles and abstracts were screened for eligibility by three authors. For any references where authors were unsure as to whether the study met the inclusion criteria, a full text of the article was obtained to aid decision-making and we ultimately used a fourth author as an arbiter if uncertainty remained. Final study inclusion screening was done by five authors and uncertainties were thoroughly discussed and sixth author was used as arbiter where necessary. Full texts of all articles that were agreed as eligible for inclusion were retrieved. Study authors were to be contacted about unclear or missing information; in the end this was not necessary. PRISMA flowchart was used to demonstrate the process.

### Critical appraisal

Three reviewers independently appraised each of the included studies using structured critical appraisal tools. Critical appraisal forms for mixed methods were piloted, such as Mixed Methods Appraisal Tool Version 2011 (MMAT-V 2011) [31] and Critical Appraisal Skills Programme (CASP) tools [33]. Both suggested tools are widely used for systematic review purposes. They have been previously standardized and validated.

Each tool was tested with two full text papers after which three reviewers agreed the best tool to work with. CASP was selected as it was easy to use and allowed a standardized approach from more than one reviewer adding clarity to the process. Any discrepancies were resolved through discussion between the three authors. Despite the fact that some studies may have had some gaps in relation to methodological quality and reporting findings (adherence rates are usually not reported etc.), through the critical appraisal they were still included as their contextually-rich details contributed to the overall narrative synthesis and answered the research question.

Papers were assessed on the basis of how appropriate the approach used was deemed to be; whether the design and methodology were rigorous, clear and reliable; whether the role of the researcher and the context were clearly described; how convincing and relevant the findings were to the aims of the study; whether the conclusion was adequate; and whether ethical considerations were coherent and clear [34].

### Risk of bias assessment

A thorough assessment of ‘risk of bias’ and methodological quality was applied to ascertain the veracity of the findings and the strength in the arguments stated in the papers.

The variety of papers (academic qualitative, academic quantitative and grey literature qualitative) demanded different assessment tools; this differentiation is illustrated in the tables presented below. Qualitative studies were assessed by two reviewers independently using a variety of methods (see Tables 1 and 3), including using CASP as previously mentioned. There were some exceptions. One research paper was a systematic review and was appraised by A MeaSurement Tool to Assess systematic Reviews (AMSTAR 2) [39] specifically designed for the appraisal of systematic reviews. Other papers were retrieved through grey literature search and appraised with the Methodological Quality Checklist for Stakeholder Documents and Position Papers (MQC-SP) [40], designed to evaluate the quality and risk of bias of the papers. The results of the risk of bias assessment were incorporated into the narratives of the review.

**Table 1:** Quality assessment of the qualitative studies

Reference	Theoretical approach		Study design	Data collection	Validity			Analysis						Ethics
	1.1	1.2	2.1	3.1	4.1	4.2	4.3	5.1	5.2	5.3	5.4	5.5	5.6	6.1
Bantry Whyte and Montgomery, 2015 [35]	+	+	+	+	+	+	+	+	+	+	+	+	+	+
Phinney et al., 2016 [36]	+	+	?	?	+	+	+	?	?	+	+	+	+	+
Van Alphen et al., 2016 [34]	+	+	+	+	+	+	+	+	+	+	+	+	+	+
King et al., 2017 [37]	+	+	+	+	+	+	+	+	+	+	+	+	+	+

+ yes/good; no/not good;? not sure/dubious

+ yes/good; no/not good; ? not sure/dubious

1. Theoretical approach

1.1 Is a qualitative approach appropriate? 1.2 Is the study clear in what it seeks to do?

2. Study design

2.1 How defensible/rigorous is the research design/methodology?

3. Data collection

3.1 How well was the data collection carried out?

4. Validity

4.1 Is the role of the researcher clearly described?; 4.2 Is the context clearly described?; 4.3 Were the methods reliable?

5. Analysis

5.1 Is the data analysis sufficiently rigorous?; 5.2 Are the data “rich”?; 5.3 Is the analysis reliable?; 5.4 Are the findings convincing?; 5.5 Are the findings relevant to the aims of the study?; 5.6 Are the conclusions adequate?

6. Ethics

6.1 How clear and coherent is the reporting of ethical consideration?

Quantitative studies assessed the risk of bias using the domains as stated in Table 2: random sequence generation; allocation concealment; blinding (participants, personnel or outcome assessors); completeness of outcome data. Judgements concerning risk of bias for each study is classified using “yes”, “no” or “unclear” indicating high, low or unclear risk of bias respectively.

**Table 2:** Quality assessment of the quantitative studies

Reference	A Selection bias	B Study design	C Confounder	D Binding	E Data collection	F Withdrawals and drop-outs
Lowery et al., 2014 [41]	+	+	+	+	+	+
McCurry et al., 2010 [42]	+	+	+	+	+	+/−
Rantakokko et al., 2017 [43]	+	+	+	+	+	+
Van Uffelen et al., 2009 [44]	+	+	+	+	+	+/−

+ strong; +/− moderate; − weak

The symbols ‘+’ and ‘-‘were used to indicate that the paper positively or negatively answered the question in each variable (Tables 1 and 3). The symbols ‘+’ and ‘-‘were used in Table 2 to indicate whether the variables were observed or not in the papers. The symbols ‘?’ and ‘+/−‘were used to demonstrate that the explanation of that particular variable was not explicit.

**Table 3:** Quality assessment of grey literature

Reference	Study characteristics				Outcomes						Study type					Decision
	A.1	A.2	A.3	A.4	B.1	B.2	B.3	B.4	B.5	B.6	C.1	C.2	C.3	C.4	C.5	+/?
Harrison et al., 2017 [38]	+	+	+	?	+	+	+	+	+	+	+	+	?	+	–	

A. Study characteristics

A.1 Leadership: Was the organisation initiated by the local community, and does it contunue to be led by the local community to meet a local need?

A.2 Place: Is the organisation defined by its link to a physical place?

A.3 Community value: Is the primary purpose of the organisation to generate economic and social value in its community through its activities and the reinvestment of profits locally?

A.4 Local returns: Does the organisation trade in goods or services as a means to being mainly independent of grants, and ultimately generating economic returns?

B. Outcomes

B.1 Health; B.2 Social care; B.3 Wellbeing outcomes; B.4 Social engagement outcomes; B.5 Community and resilience outcomes; B.6 Carer outcomes

C. Study type

C.1 Cross-sectional; C.2 Interviews; C.3 Focus groups; C.4 Observational; C.5 Experimental

D. Decision (with reasons for either inclusion or exclusion or not sure)

In other cases, such as in Gibson et al. [45] for example, there was a clear explanation of the future steps in terms of research, but it did not follow the policy making suggestions voiced by the project’s participants. In this case, although further clarification as to the purpose of the project and the expected outcomes would have been desirable, the information provided was still appropriate to be included in the review.

### Assessment of homogeneity / heterogeneity

Homogeneity was assessed in terms of study population, intervention characteristics and reported outcomes. We detected substantial clinical, methodological and statistical heterogeneity (where applicable) across included studies, therefore we did not report pooled results but instead used a narrative approach to data synthesis. We took into consideration the possible clinical or methodological reasons for this variation by grouping studies that are similar in terms of populations, intervention features or methodological features.

### Data synthesis

We conducted a narrative analysis as heterogeneity of findings was found to be high, similar to our previous work in this field [24, 28, 29]. Firstly, a preliminary synthesis was conducted to develop an initial description of the findings of included records and to organise them so that patterns across records could be identified [30]. This was followed by the iterative approach of a thematic analysis, where multiple ideas and conclusions across a body of literature are categorised into themes [27]. Data extracted from articles were entered into Table 1. Following PRISMA guidelines for reporting, extracting data involved very brief descriptive synthesis (and a full table is included in the Appendix 1 and 2).

## Findings

### Methodological quality of selected papers

Papers were assessed using a variety of parameters to ascertain their methodological quality, as presented in Tables 1, 2 and 3.

All papers were thorough in their presentation including a specific section or sections on their methodological approach, the role of the researcher, their outcomes, data gathering tools, risk of bias and analysis of potential shortcomings.

In addition, some studies reported that there was some caveat with regard to the findings due to prior level of activity (participants were already active due to other complementary activities [44], percentage of participants interviewed and time of day [44]. Taking these variables into consideration there were still common denominators that we identified in terms of barriers and facilitators to adherence to physical activity with a focus on walking.

This section highlights the common themes found in the selected papers. The focus of the findings is not to evaluate each paper but to highlight what barriers and facilitators are identified in the recent relevant literature, as well as what adherence factors where mentioned. This review enable an understanding of the current understanding of the topic and identifies gaps to lead to further areas of research.

### Common themes

The common themes section is divided into barriers, facilitators, and adherence levels. All papers addressed the barriers and/or facilitators; only five of them addressed adherences as well.

The main themes identified in the 8 selected papers were as follows:

- The use of the environment as a tool to entice physical activity [35].
- The use of walking within a wider set of activities to support people with dementia [36, 42, 44].
- The program design, from the staff perspective, to support people with dementia [35, 37].

These themes were taken into consideration in the analysis of the sections presented below.

### Barriers

#### Bio-medical reasons & mental wellbeing & physical ability

A variety of barriers were presented in this area. Firstly, the type of activity and the level of intensity can create a barrier. Van Uffelen et al. [45] pointed out that the adherence is higher in flexibility programs than in aerobic-exercise programs. A reason for this could be the required lower intensity of flexibility programs, which make them suitable for a higher proportion of the older population.

The second point was identified by the same study [44] which reported the role of health (corroborated further by Alphen et al.) [34], other commitments (family, work, volunteering, a busy life otherwise), self-motivation and environmental issues as the main barriers to adherence. Health related factors were corroborated by Rantakokko et al. [43] also linking to walking difficulties and consequently the risk of becoming home bound. The health factor is also pointing to the intensity of the walking group exercise that may be uncomfortable for the participants [43, 46].

The last point was highlighted by McCurry et al. [42] stressing depression levels and higher behavioural disruption scores as another barrier for the regular uptake of walks. These are sometimes jointly referred to as BPSD (behavioural and psychological symptoms of dementia) [41]. Depression and fluctuating mental health prevented individuals joining walks on a regular basis, and/or participants with dementia sometimes being a disruptive element to the rest of the group. Physical and mental limitations and difficulties around understanding the guidance of the organisers were also listed as another barrier by Alphen et al. [34].

#### Relationship dynamics

In certain circumstances, the need to rely on carers and family members could negatively affect the adherence to exercise [41, 42]. The challenge of joining a walking group for the first time was identified as a potential barrier, particularly for people who live alone and do not have a partner to accompany them [45]. Indeed, this may explain why some participants signed up for a walking group and then failed to attend. Moreover, Van Uffelen et al. [44] suggested that having other commitments, such as to family or work, could act as a barrier.

The flexible planning of the activities and the need to know the participants were also considered relevant to ensure adherence [42, 44] as well as the time and human resource to encourage participants to take part [37]. The inflexibility of the programs and participants not being appropriately supported could be important barriers for participation. King et al. [37] also pointed out the need to accept that participants may express a temporary or volatile disinterest in the activity, or they may just need a tailored approach.

When environments are being over medicalized [47] activities which are considered normal, can be more difficult to achieve both in terms of being able to follow instructions, and in terms of physical ability. This applies for example to collective activities, such as going for a walk together. Strong barriers were also seen in activities perceived to have a constant dementia-focus and a normalization of medicalization. Participants tended to search for activities which replicated those widely accessible in the community, and as Phinney et al. [36] noted, those which provided an emotionally safe environment and where dementia was not being overstressed, although it was naturally acknowledged.

#### Socio-economic reasons & environmental issues

Studies reported that findings could be skewed due to the demographics of the participants group [44]. Van Uffelen et al. [44] acknowledged that the perception of safety (which was an important element to support adherence) could not be completely explained as the demographic included in their studies provenanced from high income countries. Thus, further reflection on the links between poverty and adherence to exercise need to be considered. Phinney et al. [36] also pointed out that accommodating weaker members / walkers is a potential barrier to attending the groups.

Not having a safe environment on either an emotional or practical level were also acknowledged as barriers [36]. The negative perception of safety, as well as conventional wayfinding signs, were also reported to deter walking [48]. Conventional wayfinding signs refers to ‘directional’ (signage that tells you which way to go), ‘confirmational’ (signage which confirms we are on the right path or we have arrived somewhere or heading towards the correct location) and ‘informational’ (signage such as flight information displays (FIDs)) which help to guide through the provision of information [49].

Environmental issues, such as inaccessible or challenging routes, were also cited as a potential barrier by Gibson et al. [45]. According to these authors, uneven paths, traffic and routes with busy roads should be avoided [45]. Further to that, Bantry White and Montgomery [35] added factors such as fear of getting lost that represent a barrier to taking up walking as a regular activity. King et al. [37] mentioned relocation of walks as a potential barrier, as the stability of the walking environment is important.

Looking specifically at the environmental issues, Van Uffelen et al. [44] stated that when considering walking as a way of active travel, there seems to be a correlation between highly dense areas and a reduction in walking as distances to amenities, shops, etc. may be too short.

Organisation of the walking groups was pointed out as another important potential barrier by Alphen et al. [34]. If the walking outings are not well thought through, carefully organised and organisers are not trained in giving helpful guidance to the participants, organization can be a barrier to adherence [34]. Relying on volunteers, rather than having funded walk organisers could also serve as a potential barrier for the longevity of walking groups, as volunteers may be unable to provide the same level of support [45].

### Facilitators

#### Bio-medical benefits & benefits related to physical ability

The benefits of walking as an activity which benefits the health and wellbeing of participants has already been established in the literature [34]. It was linked to the therapeutic impact of being outdoors [34, 42, 44, 45] which has a positive impact on both physical and mental health, as there is a positive correlation between being outdoors and a reduction in symptoms of depression. It has been reported by the authors above [34, 42, 44, 46] that seeing and feeling the benefits of walking outdoors is one of the facilitators for attendance/adherence.

#### Staff, group relationship dynamics & social aspect of walking group

Overall, the social aspect of the walking groups and welcoming carers and or family members to the walking groups has been stressed in consensus by most authors [34–37, 41, 42, 44–46]. Studies highlighted the importance of the role of staff, carers and family to support walking sessions, in terms of physical support [44] but also in terms of attitude and the focus of the intervention [36]. In this sense, Phinney et al. [36] highlighted the importance of focusing on the positives of what the individual can achieve and the role of the community to facilitate general activity rather than on the disability. Flexibility in programming, individual tailoring, and implementing social problem-solving were also seen as important to support people to continue with the programmes [44]. In addition, using humor and being familiar with the participant’s past and taste in activities were also seen as important to encourage adherence [37]. Gibson et al. [45] reported that humor served a number of functions including taking the focus away from the more physically challenging aspects of walking; facilitating the discussion of sensitive topics; and enabling the quieter members of the group to feel more engaged with the group dynamic. Another important aspect was identified by Phinney et al. [36] as training the staff organising these walks in giving meaningful guidance to the participants [34, 45]. Having an effective walk leader was regarded by Gibson et al. [45] as key to ensuring the success of walking groups and was therefore an important facilitator to attendance/adherence. Their role encompassed a wide range of responsibilities beyond just leading the walks [45]. The authors recommended that walking groups continue to receive funding so walk leaders were able to provide the level of support required for groups to operate effectively [45].

Presence and attendance of the carer, if possible, with their own exercise experience [34], has been mentioned as very important by studies [34, 41, 42, 46]. Living with a partner and/or having a carer and being accompanied by partners and / or carers to the walks facilitates the adherence and lowers the stress associated with getting ready and attending the walks in general [42, 46]. This was corroborated by King et al. [37] who added developing the social aspect of the group on the list of facilitators. Offering refreshments after the walking session has been mentioned as very helpful in terms of facilitating adherence by several authors [36, 37, 45].

Gibson et al. [45] highlighted the importance of the social support provided by the group environment both for those with dementia and their carers. For example, couples could either walk together, or separately from one other, should they wish to have some time apart, an important benefit which would serve to facilitate adherence. Indeed, Gibson et al. [45] reported that the social benefits associated with belonging to a walking group served as the main facilitator for attendance for all participants, regardless of whether they were living with dementia. The authors also recommended that groups should be inclusive and not limited just to people living with dementia [45].

#### Environmental issues & individual tailoring

Studies portrayed the importance of the presence, quality and pedestrian-friendliness of footpaths, safe physical environments, and appropriate landscapes to walk in [35]. The more advanced the dementia is, the more these aspects need consideration. Gibson et al. [45] emphasized that it was important for walking routes to be accessible to all participants, including those with mobility aids, with rest stops provided. The use of landscape and safe surfaces for walking was also mentioned by Bantry White and Montgomery [35], emphasizing the need to consult dementia patients and carers/family members from the outset, rather than an afterthought. The use of signs was also reported to be a facilitator, although further consideration is needed as convention signage (e.g. banners, signs, posters etc.) could be confusing for people at different stages of dementia, as stated above [35].

Individual tailoring of the walks was mentioned as important by several studies [35, 43], including tailoring the intensity [34–36] and frequency [44] for better adherence. Gibson et al. [45] also emphasized the importance of catering for individual needs and preferences, for example some participants preferred to walk the same route each week, while others welcomed the opportunity to walk a different route.

Phinney et al. [36] reported the importance of normalizing the environment and keeping it non-medicalized and non-focusing on dementia. Keeping the aspect of walks as normal everyday activity and keep supporting all attendees to interact and engage with each other and the outdoors environment played a positive role in adherence. Gibson et al. [45] corroborated this finding, with participants viewing the walks as an activity they engaged in to maintain their health and fitness, as opposed to regarding dementia as the main reason for attendance.

#### Participants perceptions about the walks & the program

Mentioned by Van Uffelen et al. [44]; Van Uffelen et al. [46] and Phinney et al. [36], another important facilitator is to keep up-to-date with participants perceptions, feelings and observations about the walks and the program overall. Some may want to discuss intensity, some may wish to discuss the route or environment and safety, but most wanted to share their feelings and perceptions so keeping a journal seems to be facilitating their adherence as they see their views matter and they have a recognised voice to share their views.

### Adherence rates

Adherence to walking groups has been described as crucial for having such beneficial outcomes in terms of improved mood and feelings [34, 42, 44, 46]. Even though we had not intended to explore the adherence rates themselves, some studies included in the review, reported either data about adherence or attendance rate. Adherence ranged from 47% [42] to 89% [41]. Median attendance showed in studies of Van Uffelen (2008, 2009) [44, 46], with exercise twice a week varied between 63 and 71%. However, another study [37] with exercise three times a week showed median attendance around 20% with great interindividual variability. Most of the studies did not report any data on adherence or attendance. It is important to take into consideration all factors having influence on adoption of physical activity, factors having influence on maintaining adherence to physical activity as well as risk factors of dropping out [37].

## Discussion

The benefits of walking, as an activity which benefits the health and wellbeing of participants, has been established [34, 50]. It is linked to the therapeutic impact of being outdoors [34, 42, 44, 50], which has a physical and mental positive impact, as there is a positive correlation between being outdoors and a reduction in depression symptoms. This review highlights what hinders and facilitates long-term engagement with walking groups in people living with dementia in community, addressing a gap in knowledge about these topics; assisting organisations and professionals working with people with dementia and their carers to effectively plan, implement and evaluate walking programmes in the community.

This discussion will first provide an overview of the literature found on the topic of adherence. It then discusses barriers and some proposed suggestions about methods to address these, before finally addressing the facilitators to engagement.

### Adherence

Adherence is usually described as the percentage of people who finished the entire exercise program while attendance rate means number of exercise sessions attended, divided by the number of exercise sessions offered [21]. Data from sport psychology research show that studies describe adherence in huge variation such as 3–87% [51]. In the appraised literature, several theoretical models of exercise behavior were proposed, including a general social-cognitive model [52]. Based on these concepts, we may look at “adherence behavior” as the behavior influenced by a complex interaction of personal, behavioral and environmental factors.

It turns out that in older people who have dementia, other factors related to cognitive impairment play a role, such as behavioral symptoms connected with the cognitive impairment, a higher possibility of getting lost, depression, and feelings of shame (when participating in dementia-focused activities). The role of a carer and his/her competencies, ability to motivate, time etc., also influence adherence. On the other hand, there are strong facilitators or perceived benefits which may increase adherence, such as the possibility to meet other people, to chat while exercising (e.g. during walking) and a chance to gain some refreshment afterwards, as described by Gibson et al. [45], Phinney et al. [36] and King et al. [37].

### Barriers

#### Concerns about risk

Where perceived risks of the exercise outweighed the benefits, carers are less likely to encourage or facilitate adherence to such programs [2, 18, 34, 41, 42, 45, 46, 53–58]. Examples of risk that would discourage engagement and adherence include the risk of falls associated with walking difficulties [44], getting lost and potential harm while lost [35], and the feeling of loss of an emotionally safe environment [36]. There is emerging research that offers risk assessment methods for wandering that might mediate some of these concerns and indicate where preventative strategies or personalisation could be implemented [35]. Other research suggests that the use of wearable technology that uses Global Positioning System (GPS) could provide reassurance and mediate the perceived risk [59] although the evidence in this field is still emerging.

Bantry White & Montgomery [35], Barnett et al. [60], Olanrewaju et al. [2], Van Uffelen et al. [44] and Gibson et al. [45] further suggest that the physical area in which the walking takes place is of importance when considering risk. Hazards such as uneven surfaces, navigating traffic and safety of the neighborhood (i.e. crime rates) have been reported as barriers to engagement. As such, the organisation and design of walking programs should be well considered, flexible and tailored for the needs of those taking part [2, 41, 44, 45, 53, 57, 58, 61]; this in itself can often pose a challenge. For example, stage of illness, levels of confidence and physical ability is likely to vary significantly across a group of carers and people with dementia. A proposed resolution to this is to consider pace, intensity and points for rest breaks are essential in the planning, design and organisation.

#### Personal circumstances and perspectives of carers

Carers are more likely to be women [54, 62–64] and carers who are employed or who had other family commitments were less likely to facilitate engagement with walking groups [2, 46, 57, 58, 65]. This is likely to be associated with how much time they had available for recreational activity but also the timing of the walking activity; sessions that occur during the day or after school might pose a challenge in such circumstances. This has also been reported in wider research with a suggestion that home-based programs of exercise are favoured [53, 57].

The ‘burden of caring’ might also have an impact on a carers willingness to facilitate access to the walking groups [2, 50, 53, 54, 56–58, 63, 64, 66–68]. It is known that carers often experience high levels of stress, anxiety and depression [2, 56, 64, 67]. Carers also suffer from physical and psychological illness themselves which impacts on their motivation to engage with such activity [54, 63, 64, 67, 68]. The notion of ‘obligation’ is reported as a barrier for carers when deciding to access support services more generally. Furthermore, Courtin et al. [62], Gibson et al. [45], Khalil et al. [55], Olanrewaju et al. [2], Suttanon et al. [57] & Wanless et al. [64] indicate that this might be linked to the relationship dynamics with the carer and person with dementia.

When carers found the exercise to be ‘boring’, or where the benefits of exercise were not apparent to them, their motivation and willingness to facilitate engagement and adherence to walking and exercise was affected [2, 44, 53, 57]. This suggests a need for appropriate, targeted recruitment to the exercise programs and development of engagement methods in partnership with a range of service providers and carers [45, 69, 70] which is lacking to date. This may include methods to raise awareness of the benefits of exercise and use instructors or healthcare professionals who are knowledgeable and willing to recommend exercise as part of a wider program of care [2, 64, 69]. The caring population is ageing with increasing numbers over 55 years [64] and therefore, long term illness and reduced mobility might be barriers for carers to engaging themselves.

Other evidence suggests that exercise does not help reduce challenging behaviour associated with dementia or depression symptoms [50]. However, clinical guidance and research literature indicates that short periods of light-moderate intensity exercise, such as walking, can improve the symptoms of depression and reduce stress levels [71, 72]. This further reinforces the need for the benefits of exercise to be promoted by healthcare professionals, as part of a multi-dimensional program of care and through recruitment strategies employed by walking group instructors. It also suggests the need to explore this concept through further research in this field.

This review has also highlighted that the voice of carers is not being invited and incorporated into the research in this field.

#### Co-morbidity

Co-morbidity with long term illnesses and other health-related problems are known barriers to engagement and adherence [2, 32, 44, 46, 53, 58, 63, 67–69]. People who experience walking difficulties [43, 53, 70] or who have pain associated with other illness [2, 45, 53] are often discouraged from engaging and/or continuing with such activity or might lack confidence in their abilities [2, 73].

#### Socioeconomic status

Socio-economic status and those from areas of deprivation are less likely to seek and engage with support services [62, 64, 65, 68] and this is reflected in the literature on walking and exercise programs [2, 32, 44, 73]. This could be explained by some of the ‘hidden costs’ of caring and the reported financial burden on carers [62, 64, 65, 68]. Conversely, it indicates that any walking or exercise program needs to have public transport links or be within walking distance and carry little to no cost to participants [2, 57, 73]. Some groups reported that the offer of free refreshments would encourage engagement [45, 57].

### Facilitators

#### Previous experience of exercise

Previous experience and perceptions of walking have an impact on whether carers and people with dementia would engage with walking groups [2, 53, 54, 57]. Those with positive experiences of exercise, those who were active before being diagnosed with dementia or had experienced the benefits of exercise were more likely to engage with walking groups [2, 53, 54, 57]. This resonates with other research, for example, patients with Parkinson’s who experienced improvements in their ability to complete daily tasks were more likely to continue with an exercise program [55].

Gibson et al. [45] reported the importance of ‘humor’ and ‘fun’ associated with exercise; walking as a recreational and social activity. This also suggests a possible ‘social aspect’ of group walking activity. The social aspect and positive reinforcement of exercise has been theorized as a possible link between adherence and exercise for people later in life when considering exercise as a learning activity [74].

#### Social benefits

Literature notes a positive effect on psychological wellbeing because of socialising with others in the same or similar position [2, 45, 53, 57, 75]. While there is some literature that indicates preference for home-based or non-group exercise due to the moderate or severe cognitive impairment associated with dementia, changeable ‘mood’ and behaviors of those with dementia [50, 53, 57]. Phinney et al. [36] indicates that the concept of ‘belonging’ in the community, citizenship, positive reinforcement and feedback from peers, relatives and instructors has been shown to facilitate engagement and adherence to group exercise and walking groups [18, 36, 75].

#### Expectations about outcomes

Research suggests that keeping a record of exercise, any improvement associated with exercise or use of wearable technology to evidence ‘achievement’ and ‘progress’ can facilitate engagement and adherence to such activities [57]. The emerging use of smartphones, fitness applications and accelerometers could provide a method by which individuals could monitor their achievement and any improvement [18, 44, 56, 58, 59, 76]. Van der Wardt et al. [18] also suggest exploring the potential of ‘gamification’ for making exercise enjoyable and fun.

Positive feedback, prompting and consistent reassurance from carers and/or instructors have been associated with engagement, adherence and improved confidence with walking [55]. Khalil et al. [55] explored exercise adherence in patients with Parkinson’s disease and suggested that patients who experienced improvements in daily function were more likely to continue with exercise; this could also be true for people with dementia and their carers. While some people with dementia do not engage with walking or exercise due to a lack of confidence in their abilities [53], exercise programs that help to build confidence, allow people to see the benefits of outcomes at an individual pace and intensity are shown to be of benefit [2, 44, 53, 55].

Interestingly, van der Wardt et al. [18] found that ‘goal setting’ from the outset did not appear to improve engagement or adherence in those with mild cognitive impairment and dementia suggesting that it could be the ‘emerging’ benefits and success that has a greater impact.

#### Living arrangements and relationship dynamics

Carers who co-habit with the person with dementia were more likely to facilitate engagement with walking and exercise as part of a ‘routine’ [46, 53]. Having a spouse as a carer or ‘walking as a couple’ has been shown to be a facilitator for engaging with walking groups [18, 45, 57]. This is likely since the primary carer lives in close vicinity or ‘with’ the care recipient and there is an element of convenience, but this is likely to link with the social aspect of walking groups as well [45].

#### Tailoring and personalisation

The concept of ‘tailored’ options in timing, intensity and frequency was of importance [32, 36, 45, 57] and other literature on the topic of exercise agrees, with carers reporting that walking and /or exercise as part of a routine or ‘program of care’ is well received [45, 64, 67, 68] as was the opportunity to engage with service users/providers with knowledge of the benefits of exercise [45, 75]. This suggests that walking groups could be offered in partnership or alongside other services and support groups [2, 18, 34, 44, 57, 70]. Literature also indicates that programs that were organised by trained, knowledgeable instructors or those recommended by healthcare professionals were more likely to attract engagement and continued adherence [64, 67, 68].

The nature of dementia as an illness can mean that routine, custom and habit play and important role in the care of the individual [76, 77]. Thus, a facilitator to engagement and adherence in programs of exercise or walking groups is more likely if they are embedded in the routine of the individual and/or carer [2, 53, 57]. Therefore, the organisation and design of such activity needs to consider this as a factor.

Another important area is communication and capacity of the facilitators/trainers to communicate appropriately with older persons living with dementia. The need to involve people with dementia in research, particularly around their experiences of communication, is evident and especially important for facilitating person-centered care, strengthening social relationships, and informing training programs [77]. Strategies were recently summarized by Alsawy et al. [77] as practical techniques and strategies reflecting interpersonal characteristics and need for people with dementia to have more active involvement was highlighted.

#### Design and location

Programs need to be flexible in location, timing, frequency and intensity or the possibility for walking programs to be based in community centers, NHS premises, enclosed gardens or community gyms [2, 45, 61]. In doing the latter of these, and with effectively trained instructors, walking groups/programs might also be used as much needed respite for carers [45]. Conversely, Suttanon et al. [57] suggest the potential benefit in offering ‘mixed programs’ whereby exercise could be home-based and outside in walking groups to allow flexibility for carers who have work or family commitments.

Gibson et al. [45] indicate that walking routes that are deemed as physically safe are more likely to recruit members to the group, as are appropriately trained instructors or those led by healthcare employees/professionals; this is likely associated with the desire to access support from healthcare providers in a convenient location [2, 53, 57, 58, 60].

There is mixed evidence about whether any walking route should be ‘changed’, how frequently or if at all [45] and this might be associated with personal need, preference or stage of illness (and therefore links with the need to tailor and ‘personalise’ walking programs and/or routes). The need to make the best use of sensory aspects in the environment and changing seasons is reported to be of benefit to people with dementia [45, 78]. For example, walking in gardens where there are plenty of flowers or water features during the summer months and trees during autumn. However, there should be consideration of risk versus benefit for everyone; a balance between the barriers and facilitators. Alternatively, Gibson et al. [45] suggest that retaining the same route and type of exercise for each occasion helps to build confidence and might reduce ‘wandering’ or risk of wandering [45] and that it reinforces ‘routine’ and ‘habit’ which has been identified as a facilitator.

Pace and intensity need to be flexible and there should be safe place to ‘rest’ along any walking route [2, 45, 53]. Conversely, the size of the walking group and approach to inclusivity (e.g. walking at the pace of the slowest person) is a factor in facilitation and adherence [36, 44].

There are various suggested locations for group walking/exercise with many benefits reported for both indoor and outdoor activity [78]. King et al. [37] suggest that indoor walking and exercise groups could be viewed as safer for those with lower cognitive functioning or confidence and can be used as part of a respite package for carers such as that reported in Gibson et al. [45] where a local Day Care Centre was used. Enclosed gardens, NHS premises, community gyms and public outdoor areas have also been reported as suitable locations for such activity [37, 78] but these require suitable staffing levels to support those using the service [37].

### Limitations

There is limited published research that discusses the barriers and facilitators to adherence of walking groups for people with the dementia in the community setting.

The influence of carers on the barriers and facilitators to adherence of walking groups is evident but from the articles included in our review, there is a clear gap in research that explores carer experiences and perspective. Further research into this area would provide much needed knowledge about how carers can impact on adherence to walking groups.

Of the limited evidence that is available, being a Bangladeshi, Pakistani or Indian woman is associated with a propensity to take on a caring role [64]. Hence, ethnicity could play a role in these carers’ willingness to seek support and access available services. Further research in these communities could be recommended to explore the potential impact of ethnicity on what facilitates and hinders participation and adherence to walking group exercise.

Lamb et al. [61] reported higher recruitment and compliance in a moderate to high intensity exercise program for men with dementia. It was also found that most of the female participants lived alone and thus, lacked the prompts and motivation to engage with exercise. This suggests gender and ‘living alone’ as a potential barrier to exercise although this has not necessarily been reported in the literature on walking groups. However, further research that explores any potential links with gender could be advisable.

This review focused on only literature published in English. Given the lack of evidence, further searching could be done in other languages. Moreover, it is likely that more walking -groups or similar activities exist but are not regularly or robustly evaluated and published about. There is therefore scope to add to this literature through evaluating existing programs. Lastly, the analysis focused on a barrier and facilitator framework; however, many factors could be both of these depending on how they are perceived or evaluated (e.g. environment). This indicates that evaluations of such programs need to account for differing perspectives.

## Conclusions

This systematic review of literature has highlighted known barriers and facilitators to adherence to walking groups type of exercise for people living with dementia in community. Carers’ willingness to engage, their own circumstances, their perspectives and experiences of exercise seem to play a key role in facilitating participation and adherence. However, there is little research that explores this. The design, location and organisation of exercise programs, and therefore, walking groups can be significant for the facilitation or prevention of engagement and adherence and this reflects the need for such activity to be part of a wider ‘program of care’, tailored to the needs of the individual that is also flexible and convenient. Knowledgeable and well-trained instructors or healthcare professionals are recommended as group exercise leaders.

Barriers to engagement and adherence include socioeconomic status, carers’ personal circumstances and perspectives. Further research may be conducted into carer’s experiences and perspectives and the role smartphone applications and wearable technologies can play. Approaches to raising awareness of the benefits of exercise through the recommendation of health care professionals may be explored.

## Figures and Tables

**Fig. 1:**
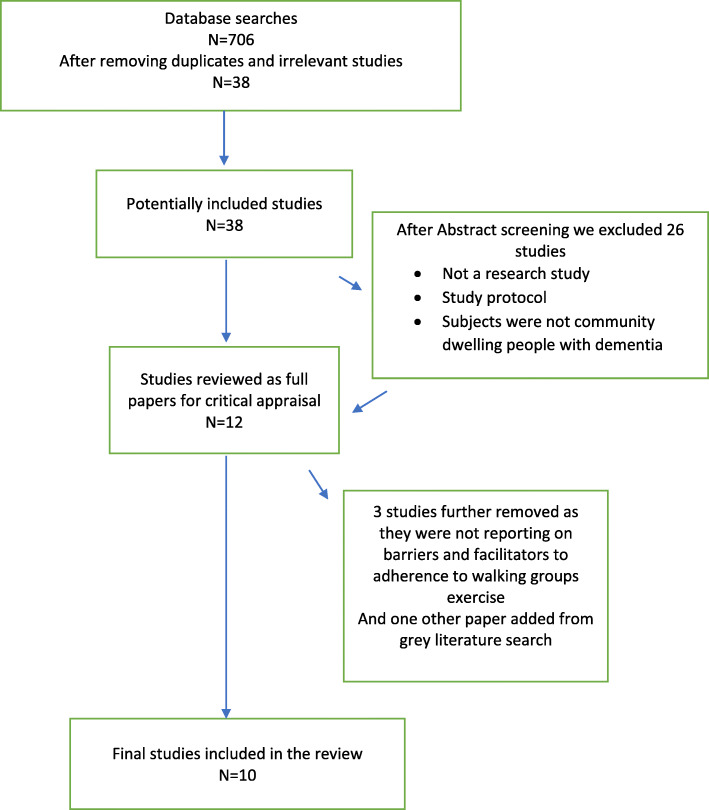
Flowchart

## Data Availability

The datasets used and/or analyzed during the current study are available from the corresponding author on reasonable request.

## Appendix 1

**Table 4:** Data extraction table for included studies

**Author(s) and year**	**Study design**	**Aim of the study**	**Type of intervention**	**Sample details**	**Main barriers**	**Key facilitators**	**Adherence data**
van Uffelen et al. 2009 [44]	RCT with a factorial design	To examine feasibility of regular moderate-intensity walking program, to assess association of exercise attendance and cognition	1 year, twice a week, 60 min, moderate intensity walking program vs. low intensity activity program	122	Lack of interest Weather Walking difficulties Health-related problems	Keeping up to date with participants’perceptions about the program and how they are coping with exercise intensity Attending at least one session – trying exercise	Median attendance 71%
van Uffelen 2008 [46]	Double blind randomized placebo-controlled trial	To examine effect of aerobic exercise or vitamin B supplementation on cognitive function	1 year, twice weekly, group based, moderate-intensity walking program vs. low intensity placebo activity program and vitamin B supplementation or placebo	152	Illness Too busy Location too far Uncomfortable intensity Health-related problems	Living with a partner	Median session attendance 63%
Bantry White and Montgomery 2016 [35]	Mixed-methods study	Wandering, getting lost and hence being restricted from walking can be a barrier to walking outdoors alone	Self-administered questionnaire	14 professionals	Factors associated with getting lost and of harm while missing	Ensuring safe physical environment and appropriate landscape and surfaces to walk on, schedule adverse risks objectively – safe walking assessment, tailoring walks and assessments to individual circumstances	Not reported
**Author(s) and year**	**Study design**	**Aim of the study**	**Type of intervention**	**Sample details**	**Main barriers**	**Key facilitators**	**Adherence data**
King et al. 2018 [37]	Randomized trial	To evaluate feasibility of implementing The Enhance Mobility Program	8 months, group exercise and walking (at least 20 min, at least 3 times a week)	28	Space reallocation Adequate staffing and time needed to recruit clients to participate Lower MMSE	Social aspect of group walking Refreshment offer at the end of walking session	Participation on walking program ranged 0–76 days out of 96 days with the walking program (M = 20.2, SD 19.6)
McCurry et al. 2010 [42]	Clinical trial	To examine factors associated with adherence to walking program	Walking 30 continuous mina day	66 dyads	Depression Higher behavioral disruption scores (RMBPC)	Spousal caregiver Lower perceived stress	47% participants were still walking 5 or more days a week at 6-months follow up
Lowery et al. 2014 [41]	Single blind parallel group trial	To evaluate effectiveness of a simple dyadic exercise regimen	Individually tailored progressive walking regimen, 20–30 min, at least 5 times a week	131 dyads	Low adherence levels	Carers‘involvement Overall BPSD (behavioural and psychological symptoms of dementia) lower if adherence is maintained	116 completed the trial (89%) Prescribed frequency of walks was achieved by 31% of treatment group, prescribed intensity in 53% of walks
**Author(s) and year**	**Study design**	**Aim of the study**	**Type of intervention**	**Sample details**	**Main barriers**	**Key facilitators**	**Adherence data**
Rantakokko et al. 2017 [43]	Life-Space Assessment, Self-reported ability to walk 2 km was assessed	Task modifications in walking may help community-dwelling older people to postpone life-space mobility decline		848/816/761	Walking difficulty, becoming home bound	Self reported modifications in walking, using mobility devices	
Phinney et al. 2016 [36]	Ethnographic study, participant observation	To explore how community-based programming can promote social citizenship,	Every day leisure group walk in neighborhood	15	Emotionally safe environment, overstressing dementia, medicalising/overmedicalising environment, not interacting with participants, not being able to accommodate weaker members,	Social view on the walking program – being part of the community, belonging, non medicalised atmosphere, **normal** everyday activities, keeping the focus off dementia, emotionally safe environment, outdoors & being able to observe and react to things happening around, enjoyment of each other’s company, sharing cards with public explaining aims of this particular group makes them more welcome in the community, group resting on principles of compassion and empowerment	Not reported
**Author(s) and year**	**Study design**	**Aim of the study**	**Type of intervention**	**Sample details**	**Main barriers**	**Key facilitators**	**Adherence data**
Alphen et al. 2016 [34]	Systematic review	To reveal factors that facilitate or hamper participation of dementia patients on PA	Review including also walking programs	7 studies with 39 dementia patients and 36 caregivers	Physical and mental limitations Difficulties with guidance Organization of PA by caregivers	Service providers familiar with exercise benefits Strategies to avoid health problems Convenient and personalized options of PA	Not reported
ROG HARRISON, KIM STRACHAN, SHEILA THORBURN 2017 – stirling dementia project grey lit	Grey literature – report	To evaluate the second year of a dementia friendly walking group project, to explore the attendees’ experiences of attending the walking groups.	Every day leisure group walks in urban, suburban and rural areas.	6 walking groups – 1 person with dementia and 1 carer from each group for individual interviews. Focus group interviews involved all the walk attendees and volunteer walk leaders in each walking group (numbers not reported)	Environmental issues making walking routes challenging/inaccessible Not having funded walk organisers Challenges posed by joining a walking group for the first time	Therapeutic impact of being outdoors Having an effective walk leader and ensuring funding remained in place to employ walk leaders Social support provided by the group for both people with dementia and their carers Having accessible walking routes Individual tailoring of walking routes Having inclusive/mixed groups, rather than making walks exclusively for people living with dementia	Not reported
